# Social representations of HIV and healthcare among recently diagnosed youth

**DOI:** 10.11606/s1518-8787.2024058005594

**Published:** 2024-10-11

**Authors:** Rosana Guarnieri, Fernanda Cangussu Botelho, Luís Augusto Vasconcelos da Silva, Eliana Miura Zucchi

**Affiliations:** I Universidade Católica de Santos. Programa de Pós-Graduação em Saúde Coletiva. Santos, SP, Brazil SP Brazil; II Universidade de São Paulo. Faculdade de Medicina. São Paulo, SP, Brazil Universidade de São Paulo Faculdade de Medicina São Paulo SP Brazil; III Universidade Anhembi Morumbi. São Paulo, SP, Brazil Universidade Anhembi Morumbi São Paulo SP Brazil; IV Universidade Federal da Bahia. Instituto de Humanidades, Artes e Ciências Professor Milton Santos. Salvador, BA, Brazil Universidade Federal da Bahia Instituto de Humanidades, Artes e Ciências Professor Milton Santos Salvador BA Brazil

**Keywords:** Social Representation, Stigma, Adolescent, HIV, Diagnosis

## Abstract

**OBJECTIVE:**

To understand the social representations of HIV and their repercussions for the healthcare among recently diagnosed youth.

**METHODS:**

This qualitative research was conducted within PrEP15-19, a study that analyzed the effectiveness of HIV pre-exposure prophylaxis in adolescents aged 15 to 19 years. Semi-structured interviews were conducted with nine participants, of whom eight identified themselves as gay men and one as *travesti*. All were diagnosed with HIV as this study was conducted in São Paulo and Salvador from 2019 to 2020. The interview guide covered aspects of HIV prevention and repercussions on care. Data were thematically analyzed and interpreted based on social representations theory.

**RESULTS:**

Participants reported experiences of stigma and discrimination related to their sexual orientation and gender identity and expression. Their diagnosis renewed these painful experiences as it referred to the common-sense social representations of HIV and AIDS based on precarious knowledge of HIV prevention, treatment, and transmission. Analysis of facilitators and barriers to care strongly related treatment adherence to health services welcoming people with information, support, and careful listening. Barriers were related to health services’ constraints, such as lack of privacy, professionals’ hostility, and insecurity regarding diagnosis confidentiality.

**CONCLUSIONS:**

The social representations of HIV are an important dimension of youths’ experience receiving their diagnosis, especially since it renews stories of violence, homophobia, transphobia, stigma, and discrimination. Understanding this based on youths’ narratives is an important tool to formulate public policies aimed at the needs of this age group. Therefore, building new social representations to mitigate stigma constitutes one of the most important elements to face the HIV epidemic among adolescents and youth.

## INTRODUCTION

HIV combination prevention is understood worldwide as a set of programs based on rights and scientific evidence that articulates biomedical, behavioral, and structural interventions in communities to meet the needs of people and communities regarding HIV prevention^1^. In Brazil, treatment for all people living with HIV is a biomedical intervention based on antiretroviral drugs that aims to prevent HIV transmission by achieving undetectable viral load and adherence to treatment, improvement in patients’ quality of life, and reduction in hospitalizations and infections due to opportunistic diseases^2^. However, several barriers to access and adherence to care in health services must be mitigated so treatment for all people can greatly impact and sustainably respond to the HIV and AIDS epidemic in adolescents and youth since coping with it includes issues that exceed attention to medical prevention and treatment. Fighting against it must include proposals in the social spheres of health, education, science, economics, politics, and social relationships^3^.

Various contexts have shown greater HIV and AIDS incidence, higher late diagnosis rates, and worse indicators for HIV care *continuum* among adolescents and youth than among adults^4^^), (^^5^^), (^^6^. Youth and adolescents in general find that the diagnosis of the infection includes HIV stigma, traumatic experiences, and lack of a support network, culminating in depression, anxiety, and suicidal ideation^7^ causing the postponement or difficulty in adhering to treatment^8^. Moreover, adult and young people who identify with sexual orientations and gender identity outside the cis-heteronormative standard are more vulnerable to the stigma, discrimination, and violence that negatively impacts their health^9^^), (^^10^^), (^^11^^), (^^12^. Thus, adolescents and youth who belong to sexual minorities and receive an HIV diagnosis face an even more adverse scenario. However, the scarce qualitative literature on adolescent and young MSM and trans women living with HIV has only addressed care adherence and continuity^13^^), (^^14^^), (^^15^^), (^^16^, with little attention to patients’ recent diagnosis experiences and their meaning and implications.

Based on these limitations, this study highlights how adolescents and youth made sense of receiving an HIV diagnosis according to social representations theory^17^, which consists of systems of interpretation by which people interact with the world and with others, exchange knowledge, and develop individual and collective relationships and behaviors^18^. Thus, this study aims to understand the repercussions of social representations of HIV in the care experiences of youth who were recently diagnosed with HIV considering the barriers produced over time by the social representations of HIV and AIDS, their negative aspects (associated with death, promiscuity, and guilt), and the impact of these representations on healthcare processes (testing, diagnosis, and initiation of antiretroviral therapy [ART]).

## METHODS

This study was nested in a qualitative investigation on access to services and linkage to care for HIV and sexually transmitted infections within community-based interventions with delivery of testing and HIV pre-exposure prophylaxis (PrEP) to youth and adolescents, in the municipalities of São Paulo and Salvador^a^, participants in the PrEP1519 demonstration study. The main objective of PrEP1519^19^ was to analyze the effectiveness of PrEP in adolescent and young MSM, transgender women, and *travestis* aged 15 to 19 years in São Paulo (state of São Paulo), Belo Horizonte (state of Minhas Gerais), and Salvador (state of Bahia). All youth who accessed the services following demand-building strategies^20^-communication and peer education activities regarding the study, PrEP, and combination prevention-and who tested positive for HIV were invited to participate in the component of the study regarding HIV incidence. In São Paulo, diagnosed youth began ART in the PrEP service until they were referred to a public HIV outpatient clinic. In Salvador, diagnosed youth were advised on how to access public health services to begin ART. This analysis was conducted at the São Paulo and Salvador, from 2019 to 2020.

Participants were selected by previous contact with healthcare providers and PrEP service navigators. The selection considered recent diagnosis and its possible negative outcomes to participants’ mental health following data collection. The interviews were conducted by experienced field researchers trained in HIV research and took place in locations that respected youths’ convenience and ensured their privacy. In Salvador, five in-person interviews were conducted on the premises of the PrEP service. In São Paulo, five interviews were conducted at the university or in places chosen by participants. The interviews lasted from about 60 to 90 minutes and were audio-recorded with the youths’ permission.

The interview guide covered themes related to health care, motivations for participating in the PrEP research, testing experience, treatment initiation, repercussions of the diagnosis on personal and social relationships and quality of life, and future prospects.

The interviews were transcribed and reviewed, and the analytical process was organized in two stages. Initially, extensive readings guided the construction of empirical categories that were then grouped into thematic nuclei that were synthesized into three analytical categories addressing stigma, welcoming, and barriers to care. The quotes illustrating the data from the analytical categories are followed by the participant’s pseudonyms, age, and study site.

This study was approved by the Research Ethics Committees of the School of Medicine at Universidade de São Paulo (opinion no. 3,082,360) and the Institute of Collective Health at Universidade Federal da Bahia (opinion no. 3,224,840) and follows Resolutions no. 466/2012 and 510/2016 of the Brazilian National Health Council. Youth signed informed consent forms.

## RESULTS

### Sociodemographics and living conditions

Regarding gender identity, nine participants identified themselves as men and one as a *travesti*. They were aged from 18 to 20 years. Regarding sexual orientation, seven youths referred to themselves as gay; two, as bisexual; and one, as pansexual; four declared themselves white; three, as Mixed-race; and three, as Black; five youths had completed high school, two attended high school, and three had begun higher education (Table 1).

**Table 1:** Characterization of youth participating in the PrEP15-19 study in São Paulo and Salvador, 2019-2020.

Pseudonym	Age	Race/skin color	Gender identity	Sexual orientation	Schooling	Site
Salim	18	White	Man	Pansexual	Attending secondary education	São Paulo
Tulio	18	Brown	Cis Gay Man	Gay	Complete secondary education	Salvador
Otávio	18	Brown	Man	Gay	Complete secondary education	Salvador
Rafiq	19	White	Man	Gay	Complete secondary education	São Paulo
Cristiano	19	Brown	Gay Man	Gay	Attending secondary education	Salvador
Adilson	19	Black	Cis Man	Bisexual	Complete secondary education	Salvador
Cristiane	19	Black	*Travesti*	Bisexual	Attending higher education	Salvador
Naif	20	White	Man	Gay	Complete secondary education	São Paulo
Youssef	20	Black	Man	Gay	Incomplete higher education	São Paulo
Samir	20	White	Man	Gay	Attending higher education	São Paulo

Reports on living conditions described greater constant or sporadic socioeconomic difficulties, including housing instability. Some participants were unemployed at the time of the interview, whereas others reported having transactional sex due to financial difficulties. Regarding place of residence, except for one participant from São Paulo who lived in the central region, the other participants lived in peripheral (São Paulo) or working-class neighborhoods (Salvador).

### Reenacting previous stigma and discrimination experiences upon being diagnosed with HIV

The narratives of youth about receiving their diagnosis portrayed the difficulties and intensity of this experience due to negative social representations of HIV and AIDS symbolisms that are culturally embedded and widespread in common sense. The symbolic and psychological strength of social representations referred to the fear of losing their gender identity references, which affected the meaning youth attribute to reality and impacted their self-image. Participants expressed feelings of abandonment, loneliness, fear, and rejection, along with uncertainties about their future. These emotions highlight significant personal, social, and professional consequences, particularly concerning the management of their diagnosis information.

The worsening and reenactment of stigmatization, discrimination, and violence at the time participants received their diagnosis referred to vulnerabilities related to disclosing their sexual orientation and gender identity and expression, especially to their families and schools, contexts that gave rise to these feelings.

I haven’t started treatment yet. For me, I still think it’s a lot of exposure... society, even more so knowing the place I have, that I have HIV, even more so in a very prejudiced place. I already struggle with the prejudice of being Black, travesti, and having HIV. Shady, girl (Cristiane, 19 years old, Salvador).

Participants expressed moments of disbelief upon receiving their diagnosis. In certain instances, societal perceptions linking HIV to death led to conflicting emotions, ranging from self-care and to risky behaviors. The impact of the diagnosis and prevailing social views on HIV were influenced by the information youths received, often perpetuating stigma through associations with deceased artists and family members who had AIDS. Additionally, stereotypes like the “stealthier” phenomenon, in which there’s a deliberate intent to transmit the HIV virus, also played a role in shaping their perceptions.

When she [the physician] said I had it, I really didn’t believe it. And I think it was hard to believe it because when she said I had it, there was a scene of my father dying in bed and my mother saying that I was going to die the same way my father did (Rafiq, 19 years old, São Paulo).

... People who don’t take care of themselves, who make a point of giving it on to other people, they have to be really rotten to do that, you know? (Otávio, 18 years old, Salvador).

The acknowledgment of their diagnosis by young people and their apprehension about facing stigma and discrimination from family and social circles highlighted vulnerable aspects related to homophobia, racism, and other forms of violence that preceded their diagnosis. These factors often led participants to delay seeking treatment.

Some interviewees described significant difficulty, and in some cases, impossibility, in disclosing their diagnosis to family members and partners. The tendency to idealize secrecy about their diagnosis emerged as a coping mechanism to alleviate insecurities when facing reality. Consequently, maintaining silence appeared to lessen the fear of having their gender identity associated with an HIV diagnosis. Sharing their diagnosis with partners often evoked feelings of disappointment, frustration, and powerlessness among participants.

When I told the first person I was hooking up with [about the diagnosis], they were completely different toward me. There was no future in that. Every single thing that happened regarding that, I felt like garbage. As if I were no longer capable of anything, not even of constituting anything else (Salim, 18 years old, São Paulo).

Friends, in turn, were those with whom youths managed to share their diagnosis, receiving support and greater comfort around the expected confidentiality.

I told a friend living far from me. She was the only one I told. Because she’s far away, she doesn’t know the people around me... (Cristiano, 19 years old, Salvador).

The experience of the diagnosis combined awareness and reflection on sexual relationships prior to the diagnosis, a time in which participants reported having failed to protect themselves and defined as situations in which they surrendered to pleasure and were unable to control prevention.

A few times I’ve gone into a dark room, left the club and went to the person’s house, to a motel. It depends a lot on that euphoria of the moment, you know? Hotheaded, we get it. Every time I’ve used ether during intercourse, I didn’t use a condom. Because I’m kind of high, penetration is the next thing I know. It’s sorta ‘that’s that’ and we just keep at it (Youssef, 20 years old, São Paulo).

...Sometimes you’re doing it without a condom and you don’t even imagine the person has it. It’s not in their face, and the person won’t go so far as to tell you “oh, I have something!,” you know? So, we run the risk, you know? (Otávio, 18 years old, Salvador).

Receiving the HIV diagnosis was a very difficult experience for participants to process due to the information they had about HIV, the references linked to the strength of social representations, and the weight of societal stigmas.

### Friendly health services and adherence to care as facilitators for building new life projects

Participants deemed health services as important spaces to obtain information, and teams’ service facilitated participants accepting ART and changing their understanding of HIV infections.

“So, if I take the two pills a day right, will I be fine?” He [psychologist] said: “You’ll be great. You’ll be 100%.” So, I said: “Oh, great.” Like, that made me feel good (Samir, 20 years old, São Paulo).

They reassured me, and all that. I liked what they said. It’s very good here, the people here are very kind, they welcomed me and everything (Tulio, 18 years old, Salvador).

Upon reflecting on their lives and future plans, some youths highlighted psychological care as an integral part of their treatment. It provided an opportunity to address various aspects of their mental health beyond their diagnosis, such as dealing with traumatic experiences, relationship challenges, and symptoms like anxiety and depression. Psychological services were crucial in assisting youths experiencing feelings of guilt, loneliness, fear, and uncertainty. At this critical juncture, they needed support rather than being viewed through the lens of societal misconceptions that associate HIV and AIDS with “unnatural sex” or “perversion”.

Because we are very young, to find out that we have a disease we will carry for the rest of our lives is something very heavy. And you can redesign everything, change it, say: “Damn, as of today I’m a different person, and it’s not going to affect my life at all.” (Rafiq, 19 years old, São Paulo).

### Constraints in healthcare places

Participants reported negative experiences related to health services, such as tensions around privacy, hostility from professionals, and insecurity regarding the confidentiality of their diagnosis.

I’m well taken care of. It’s just that I don’t like going there. I’m afraid people will recognize me. I get angry. If I could, I’d be rich and send someone to go there and get the medication (Cristiano, 19 years old, Salvador).

Participants also reported the negative aspects of their interactions with service workers, pointing out weaknesses in these services, such as rudeness, lack of ethics, and impoliteness.

When I found out, right away, I didn’t want to tell anyone. So, the fewer people know during their approach, the better. Not that it’s not necessary, but for three [professionals] approaching me like that right away, I think it’s very unethical (Cristiane, 19 years old, Salvador).

The challenges in accessing information and professionals’ tendency to make judgmental assumptions, blaming youths for their exposure to infection risks, were manifestations that reinforce the stigma of HIV linked to promiscuity, whether implicitly or explicitly.

Then, I went to the infectious disease specialist, and on the first day he saw me, he said: “You have HIV at your age? [...] Let me check.” Then he said: ‘Take this’... I took the paper and left the room crying (Cristiano, 19 years old, Salvador).

The delay in scheduling appointments also concerned youths due to the possibility of running out of medication.

I have nothing to complain about, just the delay to be served. It takes a long time. I’m worried because I took two of my medicine today, and it runs out in January. And the appointment is only in March (Youssef, 20 years old, São Paulo).

Despite contextual differences between study sites, participants’ narratives often mentioned negative imagery and perceived barriers regarding HIV and AIDS. Reports showed gaps in family and school environments regarding information on the subject and identified health spaces as the most appropriate for care, despite the mentioned weaknesses.

Because, for me, everything would be contagious [others]. For me, a kiss could be contagious. For me, a sneeze could be contagious. I closed myself off for the first three weeks, on the fourth, a month later. But then I went to the CTA [Testing and Counseling Center] again, I cleared all my doubts (Samir, 20 years old, São Paulo).

... I think it’s even an issue of sex education in schools... I used to have sex, penetration, with a condom, but I didn’t do it orally, even though you still expose yourself (Adilson, 19 years old, Salvador).

## DISCUSSION

The social representations of HIV constitute an important dimension of the diagnostic experience for youths, especially as they relive stigmatizing experiences of violence, homophobia, transphobia, discrimination, inequality, exclusion, and vulnerability that are loaded with suffering and are renewed by the diagnosis. The AIDS metaphors constructed at the beginning of the epidemic in the 1980s-associated with death, promiscuity, and punishment, loaded with discriminatory aspects, and stressed by the media up to the 1990s-remain strongly present in the symbolic context and in the construction of social representations. These representations disregarded human dignity and its vulnerabilities, occurring in the stigmatization, violence, and discrimination youths experienced in their families, schools, and public spaces and giving rise (as its main consequence) to the notion that these youths, learning of their HIV diagnosis, again find themselves incapable, alone, worthless, and enduring punishment.

The results in this study show that diagnosis is a difficult and intense moment marked by incredulity. Other studies with adolescents and youth in general in other contexts describe diagnosis as a stressful moment that precedes a period characterized by anger, depression^21^, and a reluctance to believe the result that leads to multiple tests^7^. Other studies with adolescents and youths^8^^), (^^22^ show how this traumatic diagnosis renews other traumatic and stigmatizing experiences related to physical, psychological, and sexual violence. This study, focused on adolescent and young MSM, trans women, and *travestis*, found, in addition to the aspects the literature has portrayed, experiences that included a history of stigma, discrimination, and violence related to gender and sexuality that impacted the management of diagnosis and initiation of ART. This finding highlights the importance of conceiving the moment of diagnosis and the initiation of HIV care for this population under potentially different facets of vulnerability.

Social representations of HIV, characterized by stigma and discrimination, play a crucial role as a mediator influencing individuals’ self-confidence and their decision to disclose their diagnosis within social circles. Therefore, the contexts of intimate relationships, family dynamics, and friendships undergo thorough scrutiny, prompting individuals to employ defense mechanisms such as silence and secrecy about their diagnosis. These strategies serve as primary protective measures against potential abandonment and rejection. This finding is aligned with the literature on adolescents and youths in general living with HIV^7^^), (^^23^ regarding their avoidance of sharing their diagnosis and/or difficulties disclosing it^24^. Participants also often mention fearing rejection after disclosing their diagnosis in their social circles^7^^), (^^25^, to which a component of guilt^26^^), (^^27^ is added to such fear and anxiety. Moreover, considering the scarcity of studies on the repercussions of HIV diagnosis in adolescents and youths belonging to sexual and gender minorities, this study contributes to the scientific literature by explaining that the possibility of disclosure to the family includes the fear of renewed discrimination and abandonment, reproducing their experiences of disclosing or discovering their non-heterosexual orientation. On the other hand, friendships offer the safest circle of disclosure as adolescent and young MSM and *travestis* can more clearly manage and anticipate which people under what circumstances would better protect their privacy in face of their previous experiences of violence and discrimination associated with gender and sexuality.

The findings in this study show that adolescents and youths lacked knowledge about HIV prevention, which was associated with failures in family and school environments. Studies with young MSM in China^15^ and the United States^16^ also show their lack of knowledge about HIV, its diagnosis, and related terms and point to gaps in public health policies regarding the dissemination of technologies to prevent infection and transmission. In this study, after diagnosis, HIV clinics are seen as safe places for obtaining information due to the precarious information on prevention, diagnosis, and treatment in other environments.

Several studies show that psychosocial support for youth living with HIV can increase the chances for the timely initiation and continuation of treatment that aims to achieve undetectable viral loads^8^^), (^^15^^), (^^16^^), (^^28^^), (^^29^. In line with other investigations^22^^), (^^29^, this research shows the need for diagnosis and ART to include psychosocial care. Studies show how the HIV diagnosis means an interruption in the plans of youths, requiring changes in their lifestyle and routine^7^ and provoking a process of resignification of their own lives and the construction of new projects for their future^30^. This underscores the critical role of psychological care within healthcare services in addressing emotional and attitudinal aspects, as well as understanding the defensive and self-blaming mechanisms shaped by societal representations before diagnosis. This therapeutic space offers opportunities to explore feelings of guilt and self-punishment, serving as fertile ground for evolving social representations through the integration of scientific knowledge into common perceptions. In this study, psychological care emerges explicitly linked to healthcare services as nurturing spaces that support deeper introspection on life in the context of diagnosis, and in relationships such as love, family, and work. This perspective reiterates the core essence of healthcare, conceived hermeneutically as a collaborative process in constructing “projects of happiness.” This process unfolds within the clinical encounter, surpassing mere technical success in intervention (in this case, ART)^31^.

All participants in this study, except for one young *travesti* from Salvador, used antiretroviral drugs and stressed the importance of psychosocial support at the time of diagnosis and beginning of treatment. However, adolescents and youth deemed access to and permanence in services as great challenges. The main barriers referred to concerns regarding their privacy and confidentiality and professionals’ hostile approach, all of which were experienced after the beginning of care in the specialized HIV/AIDS network. Thus, two distinct patterns of care experience in the interaction with professionals emerge: a welcoming disclosure of diagnosis (carried out within the scope of this research) and another that included these barriers at the beginning of treatment. Studies with youths living with HIV in other countries, such as China^15^, Malawi,^32^ and South Africa^33^, also highlight participants’ distrust and insecurity regarding secrecy and confidentiality in services due to professionals’ negative and hostile attitudes as the main barriers to their adherence to care. Other studies have found concerns regarding professionals^28^ being able to maintain confidentiality and the need to share the diagnosis in different services^30^. As in this study, other reports^29^^), (^^34^ point to negative interactions with health teams in building this bond and caring for youths. Therefore, the shortcomings in health services that hinder care, as perceived by adolescent and young MSM and *travestis*, appear to resonate with the traditional barriers faced by adolescents and youths living with HIV in sexual health settings. Investing in health policies and enhancing the relationships between health teams and recently diagnosed adolescents and youths offer a promising approach to reshaping symbolisms and cultivating new social representations of HIV. These representations can advocate for rights and compassionate care, thereby mitigating processes of stigma and discrimination. A fresh perspective on addressing the HIV epidemic can align with the advancements in scientific innovations, treatment modalities, and overall care practices. This holistic approach aims to foster resilience and inclusivity within affected communities.

## FINAL CONSIDERATIONS

The social representations of HIV in the care of recently diagnosed youths underscore the need to consider both the objective and subjective aspects of the health-disease-care continuum when formulating public policies for HIV prevention and treatment among adolescents and youth. Insufficient and often distorted information within social circles, schools, families, and digital media perpetuates social representations of HIV that continue to be strongly associated with stigmatizing and discriminatory notions of death, punishment, and promiscuity.

Furthermore, despite scientific and technological advancements, stigmas, tensions, and conflicts persist in the daily lives of young MSM and trans individuals living with HIV, who are still unfairly perceived as “promiscuous” or inherently “at risk”^35^. Addressing these tensions and negative social representations necessitates research focused on caregiving and strategies for combating stigma and discrimination. This approach aims to foster new social representations that emphasize human rights and resilience in the face of vulnerabilities.

## Footnotes

a Report of the research “Trajetórias e processos de vinculação ao cuidado para IST e HIV entre adolescentes e jovens homens que fazem sexo com homens, travestis e mulheres transexuais que participam de intervenções em comunidade para oferta de testagem e PrEP” [Trajectories and processes of linkage to care for STI and HIV among adolescents and young men who have sex with men, travestis, and transsexual women who participate in community interventions to offer testing and PrEP], carried out by the Graduate Program in Collective Health at Universidade Católica de Santos, Santos (SP), which received funding from Conselho Nacional de Desenvolvimento Científico e Tecnológico and the Secretariat of Health Surveillance of the Ministry of Health (grant 404055/2018-4).
