# Supplementary material for: Social representations of HIV and healthcare among recently diagnosed youth
Source: Rev Saude Publica. 2024 Oct 11;58(Suppl 1):6s. doi: 10.11606/s1518-8787.2024058005594 (PMC11573375; doi:10.11606/s1518-8787.2024058005594)
Supplement: Translation

# Translation: Representações sociais do HIV e o cuidado de jovens recentemente diagnosticados

**Authors:** Rosana Guarnieri, Fernanda Cangussu Botelho, Luís Augusto Vasconcelos da Silva, Eliana Miura Zucchi

**Affiliations:** I Universidade Católica de Santos. Programa de Pós-Graduação em Saúde Coletiva. Santos, SP, Brasil Universidade Católica de Santos Programa de Pós-Graduação em Saúde Coletiva Santos SP Brasil; II Universidade de São Paulo. Faculdade de Medicina. São Paulo, SP, Brasil Universidade de São Paulo Faculdade de Medicina São Paulo SP Brasil; III Universidade Anhembi Morumbi. São Paulo, SP, Brasil Universidade Anhembi Morumbi São Paulo SP Brasil; IV Universidade Federal da Bahia. Instituto de Humanidades, Artes e Ciências Professor Milton Santos. Salvador, BA, Brasil Universidade Federal da Bahia Instituto de Humanidades, Artes e Ciências Professor Milton Santos Salvador BA Brasil

**Keywords:** Representação Social, Estigma, Jovem, HIV, Diagnóstico

## Abstract

**OBJETIVO:**

Compreender as representações sociais do HIV e suas repercussões no cuidado de jovens recentemente diagnosticados.

**MÉTODOS:**

Foi realizada pesquisa qualitativa no contexto do estudo PrEP15-19, que analisou a efetividade da profilaxia pré-exposição sexual ao HIV entre adolescentes de 15 a 19 anos. Foram realizadas entrevistas semiestruturadas com nove jovens homens que fazem sexo com homens (HSH) e uma travesti. Todos receberam diagnóstico de HIV durante a inclusão no estudo realizado em São Paulo e Salvador entre 2019 e 2020. O roteiro de entrevista abordou aspectos da prevenção ao HIV e repercussões no cuidado. Utilizou-se análise temática dos dados, interpretados a partir da teoria das representações sociais.

**RESULTADOS:**

Os jovens relataram experiências de estigma e discriminação relacionadas à orientação sexual, identidade e expressão de gênero. Essas vivências trouxeram sofrimentos que foram atualizados na comunicação do diagnóstico, pois remetiam às representações sociais do HIV e aids disseminadas pelo senso comum, baseadas no conhecimento precário sobre prevenção, tratamento e transmissão do HIV. Na análise dos facilitadores e barreiras ao cuidado, a vinculação ao tratamento esteve fortemente relacionada ao acolhimento nos serviços de saúde, com informações e apoio, além de escuta qualificada. As barreiras estiveram relacionadas às fragilidades nos espaços de saúde que obstaculizam o cuidado, como falta de privacidade, hostilidade de profissionais e insegurança quanto à confidencialidade do diagnóstico.

**CONCLUSÕES:**

As representações sociais do HIV constituem uma importante dimensão da experiência diagnóstica de jovens, sobretudo por atualizar histórias de violência, homofobia, transfobia, estigma e discriminação. Sua compreensão, a partir dos discursos de jovens, constitui importante ferramenta para formulação de políticas públicas voltadas às demandas dessa faixa etária. Portanto, a construção de novas representações sociais que visem à mitigação do estigma torna-se um dos elementos mais importantes para o enfrentamento da epidemia de HIV entre adolescentes e jovens.

## INTRODUÇÃO

No plano global, prevenção combinada do HIV é entendida como um conjunto de programas baseados em direitos e evidências científicas, realizados nas comunidades que articulam intervenções biomédicas, comportamentais e estruturais e que atendam às necessidades de pessoas e comunidades em relação à prevenção do HIV^1^. No Brasil, o tratamento para todas as pessoas (TTP) vivendo com HIV é uma intervenção biomédica baseada no uso de antirretrovirais que tem por finalidades a prevenção da transmissão do HIV via alcance da carga viral indetectável e a adesão ao tratamento de modo a melhorar a qualidade de vida e reduzir internações e infecções por doenças oportunistas^2^. Contudo, para que o TTP tenha impacto em larga escala e seja sustentável na resposta à epidemia de HIV entre adolescentes e jovens, diversas barreiras de acesso e de vinculação ao cuidado nos serviços precisam ser mitigadas, tendo em vista que o enfrentamento à epidemia de HIV e aids atravessa questões que vão além da atenção à prevenção e tratamento ligados às áreas médicas. Seu combate deve estar atrelado a propostas no âmbito da saúde, educação, ciência, economia, política e relações sociais^3^.

Em variados contextos tem sido observado aumento da incidência de HIV e aids na população de adolescentes e jovens, assim como altas taxas de diagnóstico tardio e piores indicadores no cuidado contínuo da infecção, quando comparados com adultos^4^^), (^^5^^), (^^6^. O diagnóstico da infecção para jovens e adolescentes em geral está marcado pelo estigma do HIV, por experiências traumáticas e falta de rede de apoio, culminando em depressão, ansiedade e ideação suicida^7^, com repercussões no adiamento ou dificuldade na realização do tratamento^8^. Adicionalmente, sabe-se que pessoas, adultas e jovens, que se identificam com orientações sexuais e identidade de gênero diferentes do padrão cis-heteronormativo estão mais vulneráveis a estigma, discriminação e violência, o que impacta negativamente sua saúde^9^^), (^^10^^), (^^11^^), (^^12^. Assim, é possível inferir que adolescentes e jovens diagnosticados com HIV e que pertencem a minorias sexuais enfrentam um cenário ainda mais adverso. No entanto, a escassa literatura qualitativa voltada para adolescentes e jovens HSH e mulheres trans vivendo com HIV aborda apenas a vinculação e continuidade do cuidado^13^^), (^^14^^), (^^15^^), (^^16^, sendo a experiência recente do diagnóstico, seu significado e implicações menos explorados.

A partir dessas limitações, neste estudo são destacadas as interpretações da experiência do conhecimento do diagnóstico de HIV entre adolescentes e jovens, orientadas pela teoria das representações sociais^17^, que consiste em sistemas de interpretação pelos quais as pessoas interagem com o mundo e com os outros, trocam conhecimentos e desenvolvem relacionamentos e condutas individuais e coletivas^18^. Assim, teve-se como objetivo compreender as repercussões das representações sociais do HIV nas experiências de cuidado de jovens recentemente diagnosticados com HIV, considerando as barreiras produzidas ao longo do tempo, pelas representações sociais do HIV e aids, seus aspectos negativos associados à morte, promiscuidade e culpa e o impacto dessas representações no processo de cuidado da infecção (testagem, diagnóstico e início da terapia antirretroviral [TARV]).

## MÉTODOS

Este estudo integra uma investigação qualitativa sobre acesso a serviços e vinculação ao cuidado para HIV e infecções sexualmente transmissíveis (ISTs) no contexto de intervenções comunitárias para oferta de testagem e profilaxia pré-exposição sexual (PrEP) a jovens e adolescentes nos sítios de São Paulo e Salvador^a^, participantes do estudo demonstrativo PrEP1519. O Estudo PrEP1519^19^, por sua vez, teve por objetivo geral analisar a efetividade da PrEP entre adolescentes e jovens HSH, mulheres transexuais e travestis de 15 a 19 anos nas cidades de São Paulo (SP), Belo Horizonte (MG) e Salvador (BA). Todos/as os/as jovens que acessaram os serviços a partir das estratégias de criação de demanda^20^ - atividades de comunicação e educação de pares em torno da PrEP e prevenção combinada - e tiveram resultado positivo para o teste anti-HIV foram convidados/as a participar da pesquisa no braço do estudo de incidência. Em São Paulo, jovens diagnosticados/as iniciaram a TARV no serviço de PrEP até serem vinculados/as a um ambulatório de HIV da rede pública. Em Salvador, jovens diagnosticados/as foram orientados/as sobre como acessar os serviços da rede pública para início da TARV. Esta análise foi desenvolvida nos sítios de São Paulo e Salvador entre 2019 e 2020.

A seleção de participantes foi realizada por contato prévio com os profissionais de saúde e navegadores dos serviços de PrEP e levou em consideração o momento recente do diagnóstico de HIV e possíveis repercussões negativas do ponto de vista da saúde mental mobilizadas pela entrevista. As entrevistas foram realizadas por pesquisadores/as de campo com experiência e treinamento em pesquisas em HIV e em locais previamente marcados pelos/as pesquisadores, de acordo com a conveniência dos/as jovens e com condições de privacidade asseguradas. Em Salvador, cinco entrevistas presenciais foram realizadas nas dependências do serviço de PrEP. Em São Paulo, foram realizadas cinco entrevistas na universidade ou em locais escolhidos pelos/as participantes. As entrevistas tiveram duração aproximada entre 60 e 90 minutos e foram audiogravadas mediante autorização dos/as jovens.

O roteiro de entrevista abordou temas relacionados aos cuidados de saúde, motivações para participação em uma pesquisa de PrEP, experiência da testagem, início do cuidado da infecção, repercussões do diagnóstico nas relações pessoais e sociais e na qualidade de vida e perspectivas de futuro.

As entrevistas foram transcritas e revisadas, e a organização do processo analítico foi realizada em duas etapas. Inicialmente, ocorreram leituras extensivas que guiaram a construção das categorias empíricas que, posteriormente, foram agrupadas em núcleos temáticos, os quais foram sintetizados em três categorias analíticas que versaram sobre estigma, acolhimento e barreiras ao cuidado. As falas ilustrativas dos dados provenientes das categorias analíticas são acompanhadas do codinome do/a participante, idade e sítio de estudo.

O estudo foi aprovado pelos Comitês de Ética em Pesquisa da Faculdade de Medicina da Universidade de São Paulo (parecer nº 3.082.360) e do Instituto de Saúde Coletiva da Universidade Federal da Bahia (parecer nº 3.224.840) e está em acordo com as Resoluções nº 466/2012 e nº 510/2016 do Conselho Nacional de Saúde. Os/as jovens assinaram termo de consentimento livre e esclarecido.

## RESULTADOS

### Elementos de caracterização sociodemográfica e condições de vida

Em relação à identidade de gênero, nove se identificaram como homens e uma como travesti, com idade entre 18 e 20 anos; quanto à orientação sexual, sete jovens se referiram como homossexuais, dois como bissexuais e um como pansexual; quatro se declararam brancos, três pardos e três pretos; cinco jovens tinham ensino médio completo, dois estavam cursando ensino médio e três haviam iniciado o ensino superior (Tabela 1).

**Tabela 1:** Caracterização dos jovens participantes do estudo PrEP15-19 nos sítios de São Paulo e Salvador, 2019-2020.

Codinome	Idade	Cor	Identidade de gênero	Orientação sexual	Escolaridade	Sítio
Salim	18	Branca	Homem	Pansexual	Ensino médio cursando	São Paulo
Tulio	18	Parda	Homem Cis Gay	Homossexual	Ensino médio completo	Salvador
Otávio	18	Parda	Homem	Homossexual	Ensino médio completo	Salvador
Rafiq	19	Branca	Homem	Homossexual	Ensino médio completo	São Paulo
Cristiano	19	Parda	Homem Gay	Homossexual	Ensino médio cursando	Salvador
Adilson	19	Preta	Homem Cis	Bissexual	Ensino médio completo	Salvador
Cristiane	19	Preta	Travesti	Bissexual	Ensino superior cursando	Salvador
Naif	20	Branca	Homem	Homossexual	Ensino médio completo	São Paulo
Youssef	20	Preta	Homem	Homossexual	Ensino superior incompleto	São Paulo
Samir	20	Branca	Homem	Homossexual	Ensino superior cursando	São Paulo

Os relatos sobre as condições de vida convergiram para descrições de maior dificuldade socioeconômica, seja de forma constante ou mais esporádica, incluindo menções à instabilidade de moradia. Nesse sentido, alguns participantes identificaram-se como desempregados no momento da entrevista, outros referiram fazer programa devido a dificuldades financeiras. Em relação ao local de moradia, exceto por um participante de São Paulo que residia na região central, os/as demais participantes viviam em bairros periféricos (São Paulo) ou considerados populares (Salvador).

### Estigmas e discriminações vivenciados pelos/as jovens com HIV e a atualização das experiências no contexto da comunicação diagnóstica

Os discursos dos/as jovens sobre a comunicação diagnóstica da infecção por HIV retratam as dificuldades e a intensidade dessa experiência, fortemente mobilizadas pelas representações sociais culturalmente incutidas na simbologia do HIV e da aids, em seus aspectos negativos, disseminados pelo senso comum. Em seu aspecto simbólico e psíquico, a força das representações sociais remete ao medo da perda das relações identificatórias, afetando a significação que o jovem afere à realidade e, consequentemente, impactando sua autoimagem. Sentimentos de abandono, solidão, medo, rejeição e incertezas sobre o futuro foram evocados pelos/as jovens, com importantes repercussões pessoais, sociais e profissionais, em especial quanto ao manejo da informação do diagnóstico.

O aprofundamento e a atualização da estigmatização, discriminação e violência no momento do conhecimento do diagnóstico remetem a vulnerabilidades relacionadas à revelação de sua orientação sexual, identidade e expressão de gênero, sobretudo no contexto familiar e escolar, quando esses mesmos sentimentos se fizeram presentes.

Ainda não comecei o tratamento. Pra mim, ainda acho que é muita exposição... a sociedade, ainda mais sabendo o lugar que eu ocupo, que eu sou HIV, ainda mais no lugar muito preconceituoso. Eu já luto com preconceitos de ser negra, travesti e ainda com HIV, é babado (Cristiane, 19 anos, Salvador).

Sentimentos de incredulidade também se mostraram presentes na experiência diagnóstica. Em alguns casos, as representações sociais do HIV associadas à morte culminaram em desejos antagônicos, ora de se cuidar, ora de “fazer uma besteira”. O impacto do diagnóstico e as representações sociais acerca do HIV estavam atrelados às informações que os/as jovens tinham, ao estigma disseminado no senso comum, à associação com artistas e familiares que morreram de aids e ao estereótipo do “carimbador”, como alguém que desejasse transmitir propositalmente o vírus do HIV.

Quando ela [médica] falou que eu tinha, de verdade, eu não acreditei. E eu acho que foi difícil de acreditar, porque na hora que ela falou que eu tinha, veio a cena do meu pai morrendo na cama e da minha mãe falando que eu ia morrer do mesmo jeito que meu pai (Rafiq, 19 anos, São Paulo).

as pessoas que não se cuidam, que fazem questão de passar para as outras pessoas, a pessoa é muito podre pra fazer isso, sabia? (Otávio, 18 anos, Salvador).

O conhecimento do diagnóstico de HIV pelos/as jovens e o medo de sofrer estigma e discriminação nos círculos familiares e sociais enunciam as dimensões de vulnerabilidade atreladas à homofobia, racismo e outras violências sofridas anteriormente ao diagnóstico de HIV, o que os levaria a postergar o início do tratamento.

Nesse sentido, alguns/mas entrevistados/as relataram grande dificuldade ou mesmo impossibilidade de revelar o diagnóstico para familiares e parcerias. A idealização do segredo sobre o diagnóstico apareceu como via de reduzir a insegurança frente à realidade, e, nesse sentido, silenciar-se pareceu diminuir o receio de ter sua referência identificatória atrelada ao diagnóstico de HIV. Situações de compartilhamento do diagnóstico com parceiros foram, muitas vezes, marcadas por decepção e frustração, causando sentimento de impotência.

Quando eu contei [sobre o diagnóstico] para primeira pessoa que eu tava ficando, ela mudou completamente comigo. Aí não deu futuro. Cada coisa que acontecia em relação a isso, eu me sentia um lixo. Como se eu não fosse mais capaz de nada, nem de constituir mais nada (Salim, 18 anos, São Paulo).

Amigos, por sua vez, foram as pessoas com quem os/as jovens puderam compartilhar o diagnóstico, recebendo apoio e maior segurança em torno da confidencialidade.

Eu contei pra uma amiga de longe, foi pra única que contei. Porque ela está longe, não conhece o pessoal ao meu redor... (Cristiano, 19 anos, Salvador).

A experiência do diagnóstico gerou uma combinação de conscientização e de reflexão sobre relacionamentos sexuais anteriores ao conhecimento do diagnóstico, quando não se preveniram em situações de exposição, vivenciadas e definidas por eles como situações de entrega ao prazer e impossibilidade de controle da prevenção.

Algumas vezes eu já entrei em dark room, saí da balada e fui pra casa da pessoa, fui pro motel. Depende muito daquela euforia do momento, sabe? Sangue quente, a gente pega. Toda vez que eu usei lança no meio da relação, eu não usei camisinha. Porque tô muito meio que doido, quando eu vi já tá na penetração. Já tá lá e a gente continua (Youssef, 20 anos, São Paulo).

às vezes acontece de você tá fazendo sem camisinha e você nem imaginar que a pessoa tem, porque não tá na cara, e nem a pessoa vai chegar ao ponto de dizer a você ‘ah, eu tenho uma coisa!’, entendeu? Então, a gente fica pra jogo, né? (Otávio, 18 anos, Salvador).

O conhecimento do diagnóstico de HIV apresentou-se como uma experiência muito difícil para os/as jovens processarem, devido às informações que tinham sobre HIV e às referências atreladas à força das representações sociais e ao peso dos estigmas existentes na sociedade.

### Acolhimento nos serviços de saúde e vinculação aos cuidados como facilitadores para a construção de novos projetos de vida

O serviço de saúde foi identificado como um espaço importante para obtenção de informações, e o atendimento da equipe funcionou como facilitador para a aceitação da TARV e transformação do modo de entender a infecção por HIV.

“Então, se eu tomar certinho os dois comprimidos por dia, eu vou ficar bem?” Ele [psicólogo] falou: ”Você vai ficar joia. Você vai ficar 100%”. Eu peguei e falei assim: “Ah, então perfeito”. Tipo, isso que me fez sentir bem (Samir, 20 anos, São Paulo).

Eles me tranquilizaram, e tudo o mais. Gostei do que eles falaram, aqui é muito bom, o pessoal daqui é muito gentil, me acolheu e tudo o mais (Tulio, 18 anos, Salvador).

Considerando o contexto de repensar a própria vida e projetos para o futuro, alguns/mas jovens apontaram o atendimento psicológico como parte do tratamento, como possibilidade de elaboração de outras dimensões de suas vidas que afetam sua saúde mental além do diagnóstico de HIV, como vivências traumáticas, frustrações em relacionamentos amorosos, familiares e de trabalho e mesmo sintomas decorrentes do diagnóstico, como ansiedade e depressão. No atendimento psicológico, o jovem é visto como uma pessoa com sentimentos como culpa, solidão, medo, angústia e muitas incertezas; alguém que, naquele momento, necessita do outro como ponto de apoio e não de ser visto como uma pessoa que transgrediu o “sexo natural” por meio da “perversão”, características reforçadas pelas representações sociais sobre HIV e aids.

Porque a gente é muito jovem, e descobrir que a gente tem uma doença que a gente já vai carregar para o resto da vida, é algo muito pesado. Sendo que você pode repaginar tudo, mudar, falar: “Caramba, a partir de hoje eu sou uma pessoa diferente, e isso não vai afetar em nada na minha vida”. (Rafiq, 19 anos, São Paulo).

### Fragilidades nos espaços de saúde como barreiras para o cuidado

Foram citadas experiências negativas referentes aos serviços de saúde, como tensões em torno da privacidade, hostilidade por parte de profissionais e insegurança quanto à confidencialidade do diagnóstico.

Eu sou bem atendido. Só que não gosto de ir lá. Eu fico com medo de as pessoas me conhecerem. Fico com raiva. Se eu pudesse, seria rico e mandava alguém ir lá buscar [medicação] (Cristiano, 19 anos, Salvador).

Aspectos negativos da interação entre trabalhadores dos serviços e jovens também foram mencionados, apontando fragilidades no acolhimento, como grosseria, falta de ética e ignorância em distintos serviços.

Quando descobri, logo, eu não queria falar pra ninguém. Então, quanto menos pessoas souber para abordar, melhor. Não que não seja necessário, mas três pessoas [profissionais] me abordar assim logo de cara, eu acho muito pouco ético (Cristiane, 19 anos, Salvador).

A dificuldade para obtenção de informações e a pressuposição valorativa de profissionais, culpabilizando o jovem por ter se exposto ao risco de infecção, são alguns exemplos que reiteram, de forma implícita ou explícita, o estigma do HIV associado à promiscuidade.

Aí fui pro infectologista, e aí no primeiro dia que o infectologista me atendeu ele disse assim: “Você com essa idade já com HIV? [...] Mas deixa eu ver isso aí”. Aí ele disse: “Você vai pegar isso aqui”... Aí eu peguei o papel e saí da sala chorando (Cristiano, 19 anos, Salvador).

A demora no agendamento de consultas também figurou como uma preocupação dos/as jovens em relação à possibilidade de ficarem sem medicação.

Não tenho nada a reclamar, só a demora mesmo pra ser atendido. Demora muito. Eu fico preocupado porque meu remédio eu peguei dois hoje, e acaba em janeiro. E a consulta é só em março (Youssef, 20 anos, São Paulo).

Apesar das diferenças contextuais entre os sítios do estudo, o imaginário negativo e as barreiras percebidas em relação ao HIV e à aids foram comuns nos distintos relatos. Nos discursos, também foram observadas lacunas nos ambientes familiares e escolares quanto a informações sobre o assunto, e os espaços de saúde foram identificados como os mais apropriados para o cuidado, apesar das fragilidades relatadas.

Porque, pra mim, tudo podia contaminar. Pra mim, um beijo contaminava. Pra mim, um espirro contaminava. Eu me fechei nas primeiras três semanas, na quarta, um mês depois. Mas depois fui no CTA [Centro de Testagem e Aconselhamento] de novo, tirei todas as dúvidas (Samir, 20 anos, São Paulo).

... eu acho que isso é até um problema de educação sexual nos colégios... eu fazia o sexo, a penetração, com camisinha, mas no oral eu não fazia, mas você se expõe de qualquer forma (Adilson, 19 anos, Salvador).

## DISCUSSÃO

As representações sociais do HIV constituem uma importante dimensão da experiência diagnóstica para os/as jovens, sobretudo por mobilizarem experiências estigmatizantes de violência, homofobia, transfobia, discriminação, desigualdade, exclusão e vulnerabilidade, carregadas de sofrimento, que são atualizadas a partir do conhecimento do diagnóstico. As metáforas da aids construídas no início da epidemia, nos anos 1980, associadas à morte, promiscuidade e punição, ainda estão fortemente presentes no plano simbólico e na construção de representações sociais carregadas de aspectos discriminatórios, enfatizadas pela mídia até a década de 1990. Essas representações desconsideravam a dignidade humana e suas vulnerabilidades e estavam presentes em situações de estigmatização, violência e discriminação vividas pelos/as jovens nos ambientes familiar e escolar e nos espaços públicos, trazendo como principal consequência a leitura de que o/a jovem, quando conhece o diagnóstico de HIV, encontra-se (novamente) incapaz, sozinho/a, sem valor e sendo punido/a.

Os resultados deste estudo mostram que o momento do diagnóstico é difícil, intenso e marcado por incredulidade. Em outros estudos realizados com adolescentes e jovens em geral, realizados em outros contextos, o diagnóstico foi apresentado como um momento estressante, seguido de período caracterizado por sentimentos de raiva e depressão^21^, além de relutância em acreditar no resultado, podendo levar à realização de múltiplos testes^7^. Adicionalmente, outros estudos com adolescentes e jovens^8^^), (^^22^ mostram como o diagnóstico da infecção é traumático e faz que sejam revividas outras experiências traumáticas e estigmatizantes relacionadas a violências física, psicológica e sexual. Neste estudo, focado em adolescentes e jovens HSH, mulheres trans e travestis, além desses aspectos já retratados na literatura, a experiência do diagnóstico foi atravessada pelo histórico de estigma, discriminação e violência relacionados a gênero e sexualidade, impactando o manejo do diagnóstico e início da TARV. Este achado evidencia a importância de conceber o momento do diagnóstico e o início dos cuidados com o HIV para esse público nas distintas facetas de vulnerabilidade potencialmente presentes.

As representações sociais do HIV, carregadas de estigma e discriminação, também operam como importante mediador entre a autoconfiança e a revelação do diagnóstico em seus círculos sociais. Assim, os contextos dos relacionamentos afetivo-sexuais, o convívio familiar e as amizades passam por rigoroso crivo de análise, e mecanismos de defesa são acionados para amenizar o sofrimento e se fazem presentes na forma de silêncio e segredo sobre o diagnóstico do HIV, sendo as principais estratégias de proteção contra novas situações de abandono e rejeição. Este achado coaduna com o descrito na literatura sobre o não compartilhamento do diagnóstico como uma estratégia adotada pelos adolescentes e jovens em geral vivendo com HIV^7^^), (^^23^ e/ou dificuldades de divulgação^24^. O medo de rejeição a partir da revelação do diagnóstico nos círculos sociais também é frequentemente referido^7^^), (^^25^ e, além de medo e ansiedade, é acrescido um componente de culpabilização^26^^), (^^27^. Adicionalmente, considerando a escassez de estudos sobre as repercussões do diagnóstico de HIV em adolescentes e jovens pertencentes a minorias sexuais e de gênero, este estudo apresenta uma contribuição para a literatura científica ao explicitar que a possibilidade de revelação para a família é permeada pelo receio de sofrer discriminação e abandono novamente, reproduzindo a experiência da revelação ou descoberta da orientação não heterossexual. As amizades, por sua vez, são trazidas como o círculo mais seguro de revelação, na medida em que adolescentes e jovens HSH e trans conseguem manejar e antecipar com mais clareza com quais pessoas e sob que circunstâncias sua privacidade estaria mais bem protegida em relação a experiências de violência e discriminação associadas a gênero e sexualidade, já vivenciadas anteriormente.

Os achados deste estudo mostram que os/as adolescentes e jovens apresentavam um desconhecimento sobre a prevenção do HIV, associado a falhas nos ambientes familiares e escolares. Estudos realizados com jovens HSH na China^15^ e nos Estados Unidos^16^ também mostram desconhecimento sobre o HIV, o diagnóstico e os termos relacionados a ele, apontando lacunas nas políticas públicas de saúde quanto à divulgação de tecnologias voltadas à prevenção da infecção e da transmissão, no âmbito dos cuidados. Neste estudo, após o diagnóstico, os centros de saúde especializados no atendimento e cuidado de pessoas com HIV são vistos como locais seguros para se obter informações, considerando-se a precariedade de informações sobre prevenção, diagnóstico e tratamento em outros ambientes.

Diversos estudos mostram que o suporte psicossocial a jovens vivendo com HIV pode aumentar as chances de início oportuno e continuidade do tratamento, visando à carga viral indetectável^8^^), (^^15^^), (^^16^^), (^^28^^), (^^29^. De forma consoante a outras investigações^22^^), (^^29^, os resultados desta pesquisa mostram a necessidade de o cuidado psicossocial ser parte da comunicação diagnóstica e da TARV. Estudos mostram como o diagnóstico de HIV significa uma interrupção nos planos dos/as jovens, exigindo mudanças em seu estilo de vida e rotina^7^, provocando um processo de ressignificação da própria vida e construção de novos projetos para o futuro^30^. Nesse ponto é evidenciada a importância do atendimento psicológico nos serviços de saúde como espaço para serem trabalhadas dimensões atitudinais e emocionais, compreender os mecanismos de defesa e a culpabilização paralisantes, a partir da compreensão de representações sociais que atravessam experiências anteriores ao diagnóstico de HIV. Esse espaço, onde podem ser elaboradas culpas e autopunições, torna-se um terreno fértil para que novas representações sociais sejam construídas a partir da incorporação de saberes científicos aos saberes do senso comum. Essa dimensão apareceu neste estudo, explicitamente atrelada aos serviços de saúde como espaços de cuidado fornecendo apoio para refletir sobre a própria vida de forma ampliada, não apenas em relação ao diagnóstico, mas incluindo relacionamentos amorosos, familiares e de trabalho. Aqui se recupera o sentido mais essencial do cuidado em saúde, hermeneuticamente concebido como um processo de co-construção de “projetos de felicidade” que se dá no encontro clínico, e não se restringe ao êxito técnico da intervenção, neste caso a TARV.^31^

Neste estudo, à exceção de uma jovem de Salvador, todos estavam fazendo uso de antirretrovirais e realçaram a importância do suporte psicossocial no momento do diagnóstico e no início da TARV. No entanto, o acesso e a permanência nos serviços são trazidos pelos/as adolescentes e jovens como englobando grandes desafios. As principais barreiras identificadas pelos/as jovens referem-se a preocupações com a privacidade, confidencialidade e hostilidade na abordagem dos profissionais, todas vividas após início do cuidado na rede especializada em HIV/aids. Dessa forma, é possível identificar dois padrões distintos de experiência de cuidado na interação com profissionais: um acolhedor no contexto da comunicação diagnóstica, a qual foi realizada no âmbito da pesquisa, e outro em que essas barreiras foram identificadas ao iniciar o tratamento. Estudos realizados com jovens vivendo com HIV em outros países, como China^15^, Malawi^32^ e África do Sul^33^, também destacam desconfiança e insegurança quanto a sigilo e confidencialidade nos serviços, em decorrência de atitudes negativas e hostis por parte de profissionais, como principais barreiras de vinculação ao cuidado. Outros estudos também identificaram preocupações com relação ao sigilo por parte dos profissionais^28^ e à necessidade de compartilhar o diagnóstico em distintos serviços^30^. Assim como neste estudo, outros relatos^29^^), (^^34^ apontam experiências negativas de interação com a equipe de saúde atravessando a construção desse vínculo e, consequentemente, do cuidado dos/as jovens. Portanto, as fragilidades dos serviços de saúde que aparecem como obstáculos para o cuidado na perspectiva de adolescentes e jovens HSH e travestis parecem dialogar com barreiras clássicas vivenciadas por adolescentes e jovens vivendo com HIV nos espaços de saúde sexual. Acredita-se que o investimento em políticas de saúde e o fortalecimento dos vínculos da equipe de saúde com os adolescentes e jovens recentemente diagnosticados sejam um caminho frutífero para a reconstrução de simbolismos e, portanto, para a construção de novas representações sociais do HIV, que possam expressar direitos e cuidado e mitigar processos de estigmatização e discriminação. Paralelamente à evolução das inovações científicas, das formas de tratamento e do cuidado em geral, um novo olhar pode ser lançado sobre o enfrentamento da epidemia do HIV.

## CONSIDERAÇÕES FINAIS

As representações sociais do HIV presentes no cuidado de jovens recentemente diagnosticados mostram a importância de serem observadas as dimensões objetivas e subjetivas do processo saúde-doença-cuidado na formulação de políticas públicas para adolescência e juventude voltadas à prevenção e ao tratamento do HIV. A insuficiência e a distorção nas informações encontradas nos grupos sociais, na escola, na família e nos meios de comunicação digitais alimentam representações sociais do HIV ainda fortemente referidas às ideias de morte, punição e promiscuidade resultantes de estigmas e discriminações. Portanto, a construção de representações sociais que visem à mitigação do estigma do HIV e que afirmem direitos é um dos pontos mais importantes para a reflexão sobre políticas e programas voltados ao enfrentamento da epidemia de HIV entre adolescentes e jovens.

Finalmente, os avanços científicos e tecnológicos coexistem com a reprodução de estigmas, tensões e conflitos no cotidiano de jovens HSH e pessoas trans vivendo com HIV, percebidos/as ainda como pessoas “promíscuas” ou “de risco”^35^. O atravessamento desses tensionamentos e representações sociais negativas requer investigações que se debrucem sobre dimensões relacionadas ao cuidado e enfrentamento de estigmas e discriminações, visando à construção de novas representações sociais com ênfase nos direitos humanos e no combate a vulnerabilidades.

## Footnotes

a Relatório da pesquisa “Trajetórias e processos de vinculação ao cuidado para IST e HIV entre adolescentes e jovens homens que fazem sexo com homens, travestis e mulheres transexuais que participam de intervenções em comunidade para oferta de testagem e PrEP”, executada pelo Programa de Pós-Graduação em Saúde Coletiva da Universidade Católica de Santos, Santos (SP), e que recebeu financiamento do Conselho Nacional de Desenvolvimento Científico e Tecnológico e Secretaria de Vigilância em Saúde, do Ministério da Saúde (processo 404055/2018-4).
